# Effect of nutrition counseling on complementary feeding practices among mothers with children aged 6–23 months in West Gojjam Zone, Northwest Ethiopia: A cluster-randomized controlled trial

**DOI:** 10.1371/journal.pone.0361976

**Published:** 2026-10-29

**Authors:** Shiferaw Birhanu, Getu Degu Alene, Yeshalem Mulugeta Demilew

**Affiliations:** 1 School of Health Sciences, College of Medicine and Health Sciences, Bahir Dar University, Bahir Dar, Ethiopia; 2 School of Public Health, College of Medicine and Health Sciences, Bahir Dar University, Bahir Dar, Ethiopia; PLOS: Public Library of Science, UNITED KINGDOM OF GREAT BRITAIN AND NORTHERN IRELAND

## Abstract

**Background:**

Appropriate complementary feeding is essential for children’s growth and cognitive development. However, these practices remain inadequate in the study area. Therefore, this study aimed to evaluate the effect of nutrition counseling on complementary feeding practices among mothers with children aged 6–23 months in West Gojjam Zone, Northwest Ethiopia.

**Methods:**

A two-arm, parallel, cluster-randomized controlled trial was conducted from June 2023 to December 2024 in rural districts of West Gojjam Zone. Thirty clusters (15 per arm) were assigned to either an intervention group (n = 401), which received Health Belief Model-based nutrition counseling by Women’s Development Army leaders, or to a control group (n = 401), which received routine education. The intervention was delivered monthly for nine months from infant age 6–8 months. The primary outcome was appropriate complementary feeding practices. Secondary outcomes included minimum dietary diversity, meal frequency, acceptable diet, and egg/flesh food consumption. Data were collected via interviewer-administered questionnaires. The intervention effect was analyzed using Generalized Estimating Equations with adjustment for potential confounders. Statistical significance was set at p < 0.05.

**Results:**

The difference-in-differences in appropriate complementary feeding practices between groups was 13.2 percentage points. After adjustment, mothers in the intervention group were nearly 2.5 times more likely to practice appropriate complementary feeding than controls [AOR = 2.49, 95% CI: (1.40, 4.42)]. Children of counseled mothers were also more likely to achieve minimum dietary diversity [AOR = 1.75, 95% CI: (1.04, 2.93)], minimum meal frequency [AOR = 1.62, 95% CI: (1.01, 2.61)], and minimum acceptable diet [AOR = 2.13, 95% CI: (1.26, 3.59)] compared with controls.

**Conclusion:**

Health Belief Model-based nutrition counseling was positively associated with improvements in appropriate complementary feeding practices, minimum dietary diversity, meal frequency, and acceptable diet. Integrating such counseling into routine maternal and child health services may strengthen child nutrition programs in similar rural settings.

**Trial registration:**

ClinicalTrials.gov (NCT05871346).

## Introduction

Appropriate complementary feeding, the timely introduction of safe and nutritionally adequate solid, semi-solid, or soft foods alongside continued breastfeeding from 6 months of age, is essential for optimal child growth and development [1]. Conversely, inappropriate complementary feeding practices, including early or delayed introduction of foods, inadequate meal frequency, and limited dietary diversity, contribute to adverse nutritional outcomes such as stunting, wasting, and underweight [2–4]. Stunting during early childhood leads to irreversible impairments in physical growth and cognitive development and increases the risk of illness and mortality [5].

The 2023 national Food and Nutrition Strategy baseline survey reported high levels of child undernutrition, with stunting at 39%, wasting at 11%, and underweight at 22%, exceeding regional and global averages [6]. A recent seasonal study in West Gojjam Zone also reported stunting ranging from 35.5% to 39.2%, wasting from 8.0% to 19.2%, and underweight from 17.0% to 24.6% [7].

To address undernutrition during the first two years of life, the World Health Organization (WHO) has developed evidence-based recommendations on infant and young child feeding (IYCF). These recommendations emphasize introducing complementary foods at 6–8 months while continuing breastfeeding and progressively increasing meal consistency, frequency, and dietary diversity as children grow [8]. Mothers are advised to provide 2–3 meals per day for infants aged 6–8 months and 3–4 meals plus nutritious snacks daily for children aged 9–23 months [8,9].

Global efforts to improve IYCF practices have been aligned with major development agendas, including the Sustainable Development Goals (SDGs), particularly Target 2.2, which aims to end all forms of malnutrition by 2030 [10]. WHO strengthened global commitments to child nutrition through the Global Strategy for IYCF in 2003 [11], the endorsement of IYCF indicators in 2008 [12], and their subsequent revision in 2021 [8]. Ethiopia adopted these recommendations and developed a national IYCF strategy in 2004 [13] and launched the Seqota Declaration in 2015, committing to end stunting among children under two years of age by 2030 [14]. Despite these initiatives, appropriate complementary feeding practices (ACFPs) remain low due to socio-cultural and behavioral barriers [15–18].

Consistent with global trends, complementary feeding practices (CFPs) remain suboptimal in many low- and middle-income countries. Evidence from South Asia has reported low prevalence of timely introduction of complementary foods, minimum dietary diversity (MDD), and minimum meal frequency (MMF) [19]. Similarly, only 13% of mothers in sub-Saharan Africa practiced appropriate complementary feeding [20]. In Ethiopia, the prevalence of ACFPs varies widely across regions, ranging from 8.5% to 56.5% [15,17,21–24].

Growing evidence demonstrates that community-based nutrition counseling (NC) interventions can improve CFPs and nutritional outcomes [25–28]. However, intervention effectiveness often depends on design, delivery strategies, and the use of behavior change theories.

The Health Belief Model (HBM) provides a well-established theoretical framework for nutrition interventions. The model posits that individuals are more likely to adopt recommended practices when they perceive their susceptibility to a health problem, understand its severity, recognize the benefits of action, and feel confident in their ability to act [29]. Integrating HBM constructs into counseling has been shown to improve maternal perceptions, feeding practices, and child nutritional status [30–32]. However, evidence on the application of HBM-based NC interventions in rural Ethiopia, particularly in West Gojjam Zone, remains limited.

Previous Ethiopian studies have evaluated nutrition education, behavior change, and communication interventions targeting complementary feeding practices, dietary adequacy, child growth, and morbidity [30,33–36]. However, these studies were limited by urban settings [36], lacked control groups [33], or did not evaluate the effect on ACFPs [34,35]. Furthermore, routine health education provided through the Health Extension Program remains largely information-based and rarely incorporates behavioral theories [13].

Despite the high burden of undernutrition and the availability of community-based platforms such as the Women’s Development Army, evidence on theory-driven NC interventions to improve complementary feeding practices in the study area remains limited. Therefore, this study aimed to evaluate the effect of HBM-based nutrition counseling on complementary feeding practices among mothers with children aged 6–23 months in West Gojjam Zone, Northwest Ethiopia.

## Materials and methods

### Study setting, trial design, and study overview

This study was conducted in the rural areas of West Gojjam Zone, Amhara Region, Ethiopia, located 389 km northwest of Addis Ababa. A detailed description of the study setting has been published previously [37,38]*.*

This study employed a two-arm, parallel, cluster-randomized controlled trial conducted from June 9, 2023, to December 30, 2024, in rural *kebeles*. Clusters were randomly allocated to intervention and control groups in a 1:1 ratio to minimize contamination across participants. Mothers with infants aged 6–8 months were recruited and baseline data were collected between June 9 and December 30, 2023. The NC intervention began immediately after enrollment and continued for nine months for each mother–child pair. Because enrollment was staggered, intervention initiation and completion dates differed across participants; however, all participants received the full intervention period. Endline assessments were conducted between October 1 and December 30, 2024, when children were approximately 16–23 months old. Accordingly, the overall trial period extended from June 2023 to December 2024.

This manuscript reports Objective IV of the trial protocol, which examined the effect of complementary feeding counseling on improving appropriate complementary feeding practices among mothers in West Gojjam Zone, Northwest Ethiopia. Findings from the baseline phase of this trial have been reported previously; however, those publications were limited to pre-intervention observational analyses and did not assess intervention effects. Previous publications examined: (1) the prevalence of and factors associated with appropriate complementary feeding practices among mothers of infants aged 6–8 months using a mixed-methods design [37], and (2) the prevalence of and factors associated with anthropometric failure using the Composite Index of Anthropometric Failure among infants aged 6–8 months [38]. In contrast, the present manuscript reports the effect of a 9-month HBM-based NC intervention on complementary feeding practices and related behavioral determinants.

### Study context

Ethiopia’s Health Extension Program (HEP), launched in 2003, is a community-based strategy that delivers preventive and promotive health services through health extension workers (HEWs) and the Women’s Development Army (WDA) [39,40]. Child nutrition, including exclusive breastfeeding, complementary feeding, nutritious meal preparation, and vitamin A supplementation, is a core component of the HEP [41]. HEWs, in collaboration with *kebele* administrations, select and train model families, who then share knowledge with neighboring households. WDA leaders, chosen from these families, serve as unpaid community agents linking households with HEWs and facilitating participatory learning [42]. Evidence shows that nutrition interventions involving WDA leaders can improve complementary feeding practices [35,43]. In this study, 30 WDA team leaders (two per intervention cluster) delivered NC, with approximately one leader per 13–14 participants. Their role was to promote appropriate feeding behaviors and challenge negative community norms.

### Study population and eligibility criteria

The study population comprised mothers with infants aged 6–8 months residing in randomly selected *kebeles* where baseline data were collected. Eligible participants were those who had resided in the study area for at least six months before enrollment. Eligibility was assessed during the baseline survey, as described previously [37,38].

### Participant recruitment

Detailed baseline sampling and recruitment procedures have been published previously [37,38]. Mothers with infants aged 6–8 months were identified using immunization registration records and screened through house-to-house visits to confirm eligibility and willingness to participate. All eligible mothers in selected clusters were recruited before intervention initiation.

### Participant flow

Of the 830 mothers with infants aged 6–8 months who were screened for eligibility, 802 (96.6%) were enrolled between June 9 and December 30, 2023, across 30 randomly selected clusters (15 per arm). Twenty-eight mothers (3.4%) were excluded at baseline for not meeting the inclusion criteria. A total of 401 mothers were allocated to the intervention arm and 401 to the control arm. The flow of clusters and participants is presented in the CONSORT extension flow diagram (Fig 1, S1 Appendix).

**Fig 1:**
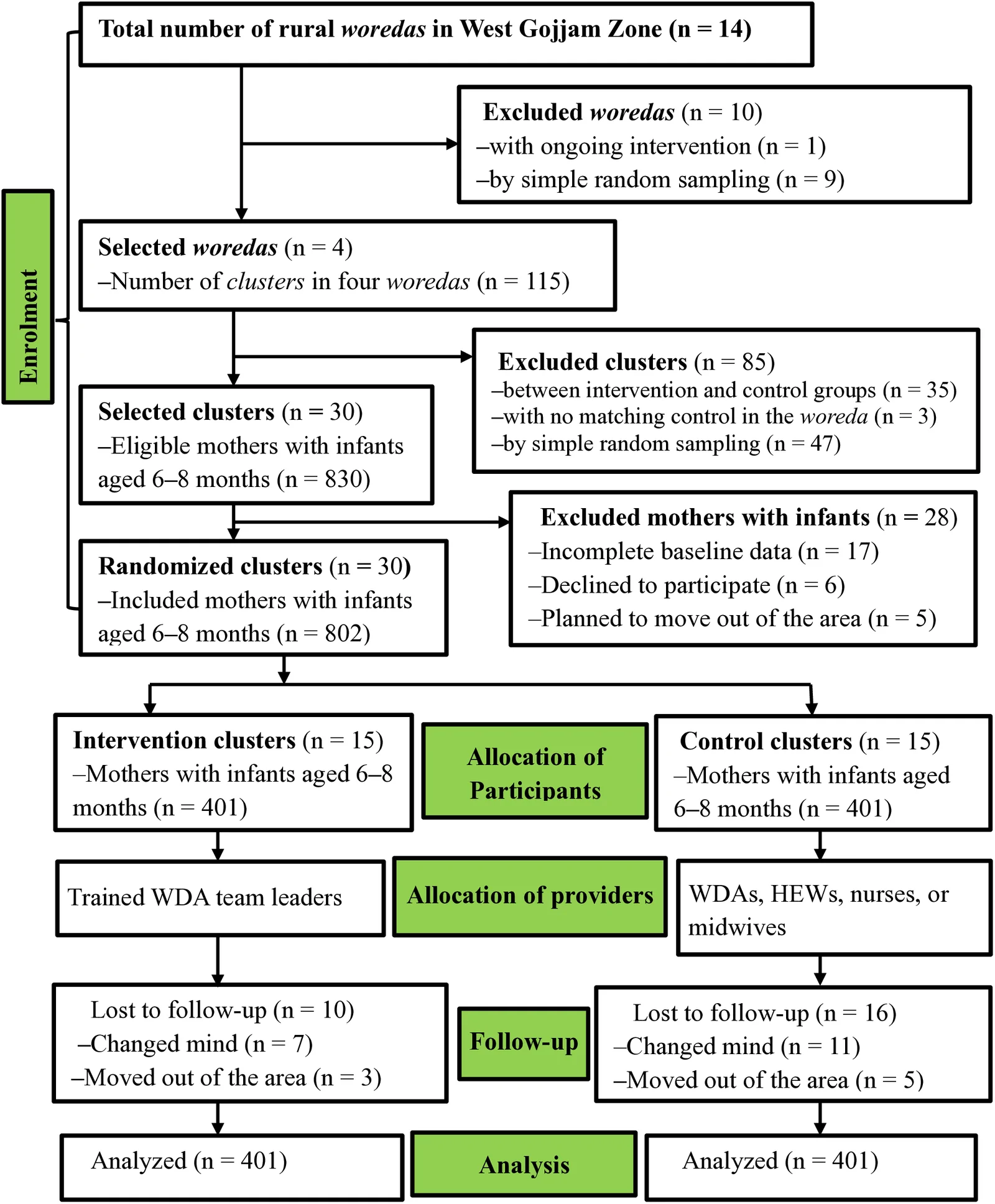
CONSORT flow diagram showing cluster selection, participant recruitment, and randomization in the trial. SRS. Simple random sampling; WDA. Women’s development army; HEWs. Health extension workers.

### Outcome measures

The primary outcome of the study was ACFPs among mothers with children aged 6–23 months, assessed at endline following the 9-month NC intervention. ACFPs were defined according to WHO- recommended IYCF indicators, requiring that TICF, MDD, MMF, and EEF consumption criteria were all met. Secondary outcomes included individual complementary feeding indicators: MDD, MMF, MAD, and EFF consumption at end line. Exploratory outcomes included HBM constructs (perceived susceptibility, severity, benefits, barriers, cues to action, and self-efficacy), maternal knowledge, and attitudes toward ACFPs. Only the primary outcome was subjected to confirmatory hypothesis testing. Secondary and exploratory outcomes were analyzed without adjustment for multiple comparisons and were interpreted as supportive findings.

### Sample size determination

The sample size was calculated based on the primary outcome, appropriate complementary feeding practices (ACFPs), using G*Power version 3.1.9.2. The initial estimation was performed at the individual level using Fisher’s exact test for independent proportions, with baseline proportion (p1) = 0.0976 [22] and an expected post-intervention proportion (p2) = 0.2176. This 12-percentage-point improvement was derived from a prior cluster-randomized trial in West Gojjam Zone, Ethiopia, where the intervention delivered through community-level actors increased minimum dietary diversity from 8% to 21% and improved other complementary feeding indicators by 10–23 percentage points [44]. A two-sided alpha (α) of 0.05 and power (1–β) of 85% were selected to ensure adequate power, assuming equal allocation between intervention and control groups. This yielded a minimum sample size of 356 participants.

To account for clustering, the design effect (DE) was calculated using DE = 1 + (m − 1) × ICC, where m = 31 is the average cluster size and ICC = 0.03 [45]. The resulting DE of 1.90 increased the minimum required sample size to 676 participants. After allowing for 10% potential loss to follow-up, the final minimum sample size was 744 participants.

Although the calculated minimum was 744, all eligible mothers with children meeting inclusion criteria were enrolled during the baseline survey [37], resulting in a final sample of 802 participants (401 per arm), exceeding the minimum requirement and enhancing the precision of the study estimates.

### Sampling, randomization, and allocation

The study employed a multistage cluster sampling design, the details of which have been described elsewhere [37,38]. Four rural woredas (*Dembecha Zuria, Jabitehenan, Burie Zuria, and Yilmana Densa*) were selected using simple random sampling, and *kebeles* were used as clusters. Eligible mothers with infants from selected clusters were recruited through complete enumeration using predefined eligibility criteria. The required number of clusters was determined using a standard formula [46].


$$\begin{array}{c} \text{C}=\;\text{1}\;+\;\frac{{\left(\text{Z}\alpha /\text{2}\;+\text{Z}\beta \right)}^{\text{2}}\;[\;{\text{P}}_{\text{0}}(\text{1}-\;{\text{P}}_{\text{0}})/\text{n }+\;{\text{P}}_{\text{1}}(\text{1}-\;{\text{P}}_{\text{1}})/\text{n }+\;{\text{K}}^{\text{2}}\;({\text{P}}_{{\text{0}}^{\text{2}}}+\;{\text{P}}_{{\text{1}}^{\text{2}}})]}{{\left({\text{P}}_{\text{0}}-\;{\text{P}}_{\text{1}}\right)}^{\text{2}}} \end{array}$$

Where C is the number of clusters required per arm; P₀ is the proportion of mothers practicing appropriate complementary feeding before the intervention (8.5%) [15]; P_1_ is the expected post-intervention proportion (18.5%); *n* refers to the average number of participants per cluster (assumed to be 31); and *k* represents the coefficient of variation between clusters, which was assumed to be 0.5.

For the cluster-level calculation, an expected improvement of 10 percentage points (P₀ = 8.5%, P_1_ = 18.5%) was assumed. Although a 12-percentage point improvement was used in the individual-level sample size calculation, a slightly smaller effect size was applied at the cluster level to provide a more conservative estimate and to account for potential between-cluster variability, as recommended in the design of cluster-randomized trials [47]. This approach helps to avoid overestimation of statistical power. Because no prior studies provided an estimate for the coefficient of variation (k) of ACFPs between clusters, a value of 0.5 was assumed. Based on these parameters, the calculated number of clusters per arm was 14.9, which was rounded up to 15. One additional cluster was added to each arm to address the limitations of the normal approximation when the number of clusters is small [46]. Accordingly, a total of 30 clusters (15 per arm) were selected.

To minimize contamination between study groups, nonadjacent clusters were selected [48]. The selected clusters were then randomly assigned to either the intervention or control group using 1:1 simple randomization. Cluster randomization was conducted jointly by health extension, nutrition, and maternal and youth officers of each *woreda*. Although cluster allocation was conducted in the presence of local health officials, these officials were not involved in identifying or enrolling participants. Recruitment procedures were identical across intervention and control clusters to minimize selection bias (Fig 2).

**Fig 2:**
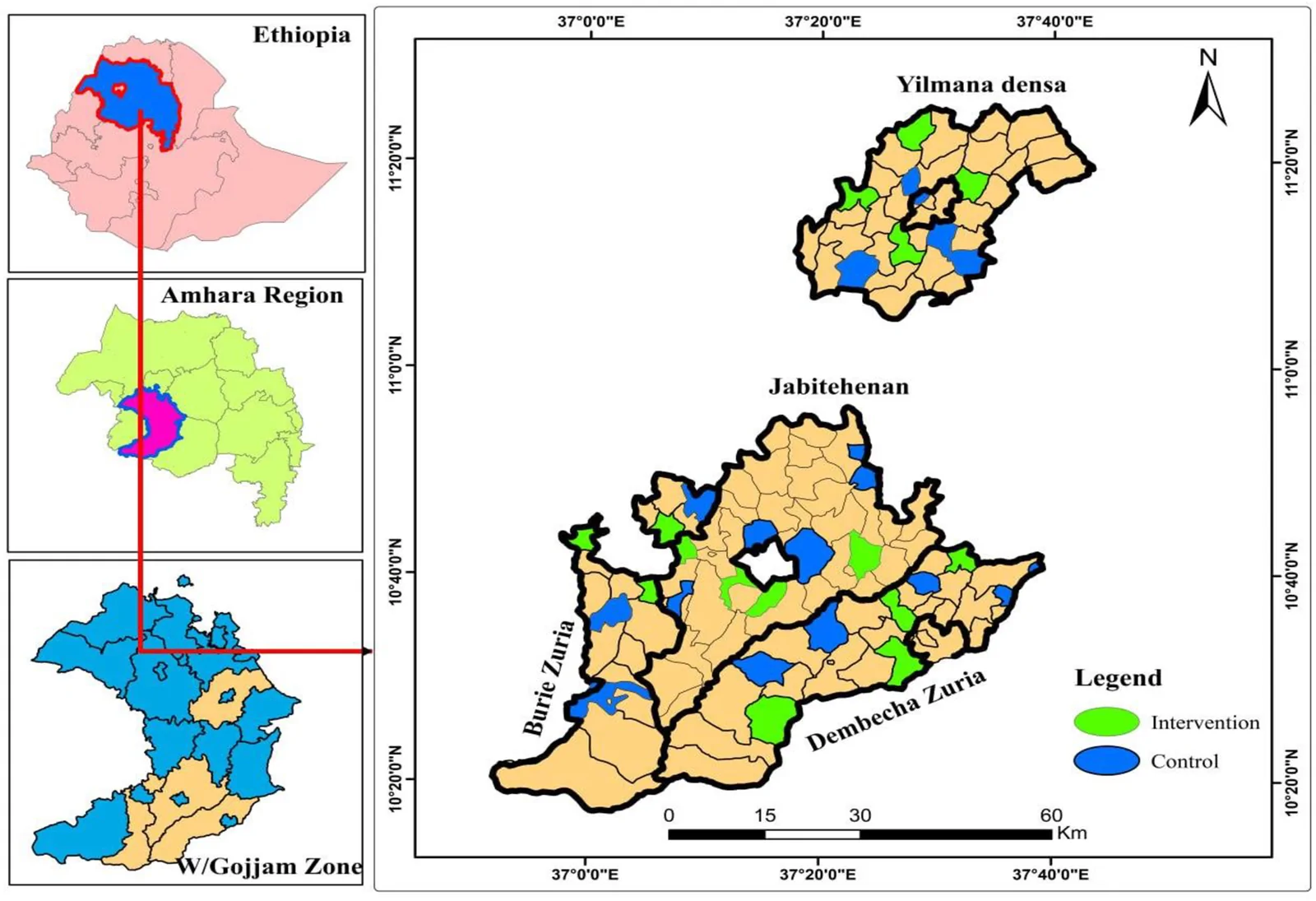
Distribution of study clusters across four rural *woredas* in West Gojjam Zone, Northwest Ethiopia. The map was created by authors using ArcGIS 10.7 software based on freely available government boundary shapefiles.

### Intervention, counsellors, and fidelity

The intervention consisted of individualized, home-based NC grounded in the HBM [29]. The counseling guide was developed based on the 2021 WHO IYCF recommendations [8] and the 2021 IYCF Counseling Trainers’ Guide [49]. The intervention was delivered by 30 WDA team leaders (2 counselors per cluster, each managing 13–14 mothers with infants on average) with prior experience in maternal-child nutrition programs. All counsellors had complementary feeding counseling-related backgrounds and received training, including a three-day pre-intervention course and a one-day refresher session four months later.

Training covered recommended IYCF practices through lectures, simulated counseling sessions, and role-plays. Content included timely introduction of complementary foods; preparation of thick and enriched porridges; energy-dense foods; iron and vitamin A-rich foods; dietary diversity; feeding frequency; egg and/or flesh food consumption (EFF); hygiene; responsive feeding; and feeding during and after illness. Counseling skills such as active listening, problem-solving, and confidence-building were emphasized, and pre- and post-training evaluations assessed counselors’ knowledge and skills.

Home-based counseling was provided for nine consecutive months following each participant’s enrollment. Sessions lasted 40–60 minutes and were conducted on weekends or religious holidays. Mothers received an illustrated counseling guide for home use; those unable to read were assisted by literate family members. HBM constructs, including perceived susceptibility to and severity of child undernutrition, and benefits and barriers to recommended feeding practices were integrated throughout the sessions [29] (Fig 3). Four MPH-trained supervisors monitored counseling delivery through monthly field visits and direct observation across all intervention clusters.

**Fig 3:**
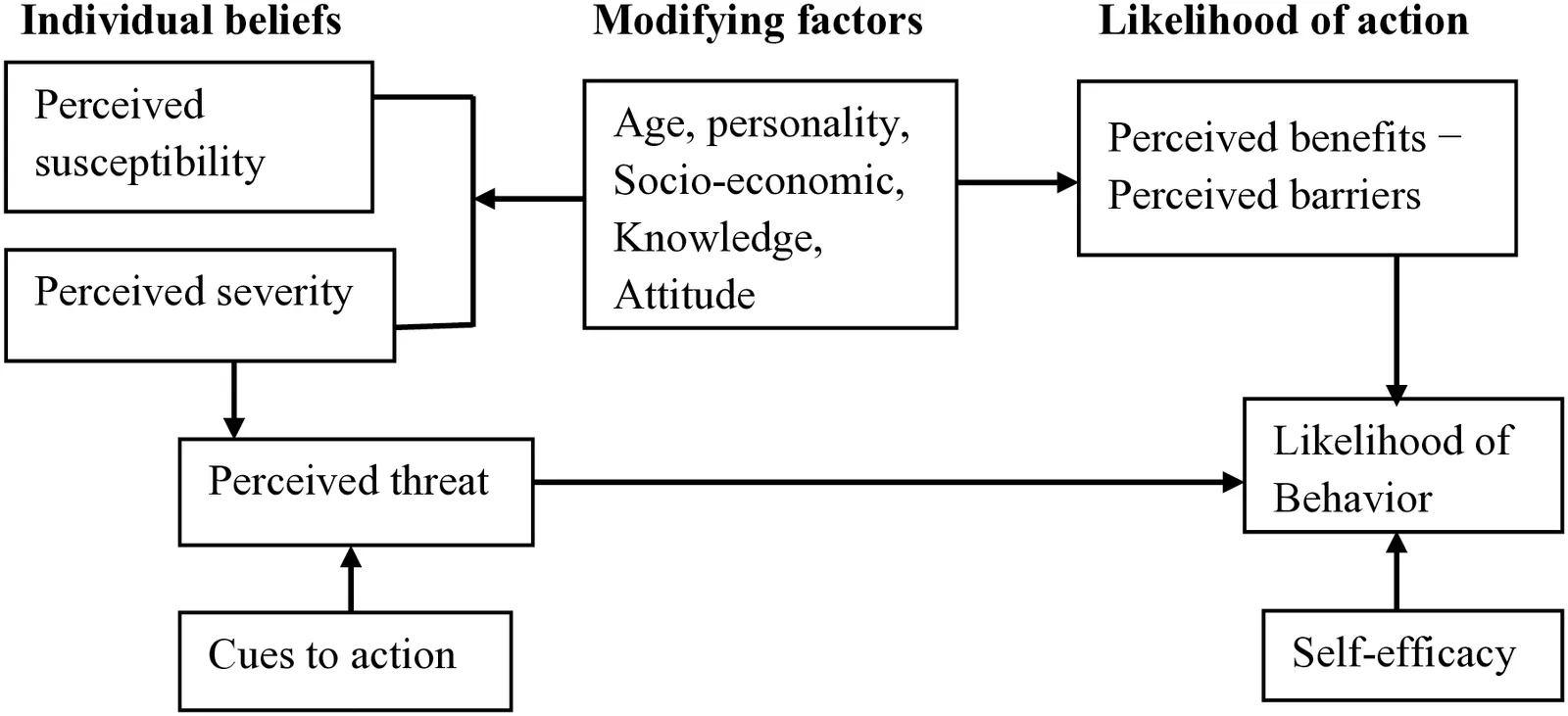
Conceptual framework of HBM-based nutrition counseling and its influence on complementary feeding practices, adapted from the HBM (Glanz et al., 2015). − . Minus.

Intervention fidelity was assessed using criteria developed by the investigators and guided by the best practices and recommendations from the National Institutes of Health (NIH) Behavior Change Consortium for enhancing treatment fidelity [50]. The checklist evaluated five domains: the intervention design, training of WDA team leaders, delivery of the NC, participants’ receipt of the intervention, and enactment of treatment skills. Intervention design fidelity was ensured through theoretical grounding from the literature, use of a counseling guide specifying content, dose, and intensity, and selection of non-adjacent clusters (*kebeles*) to minimize information contamination. An equal number of clusters were assigned to the intervention and control groups to maintain balance.

Counselor training fidelity was evaluated based on adherence to the training protocol described in the intervention section. Pre- and post-training assessments were administered to evaluate the counselors’ knowledge and skills related to the intervention.

The delivery of NC was assessed in randomly selected counseling sessions using a yes/no rating system to assess key components. These included adherence to core items of the NC guide, completeness of content delivery, duration and frequency of counseling, preparedness, and the counselor’s ability to respond appropriately to questions.

Receipt of the intervention was evaluated through participant interviews assessing understanding of key counseling messages, knowledge of minimum feeding frequency, awareness of EFF, ability to demonstrate food preparation, and confidence in applying recommended practices.

Enactment fidelity was assessed through direct observation of participants’ ability to prepare diversified complementary foods, practice responsive feeding, and demonstrate appropriate hygiene behaviors.

During each home visit, counselors assessed CFPs using the Job Aid: Food Intake–6–24 Months [49], which includes 10 key dietary intake and feeding behavior indicators. Using a client-centered approach, counselors identified existing feeding practices, assessed available household resources, and prioritized two to three counseling topics based on identified gaps. Mothers practicing recommended behaviors received positive reinforcement, whereas tailored counseling was provided to address areas needing improvement. Age-specific daily energy requirements were also highlighted [51]. Intervention delivery fidelity was maintained across clusters, with minor scheduling variations due to staggered enrollment.

Mothers in the control group received routine counseling services during antenatal care, postnatal visits, and immunization sessions. This counseling covered continued breastfeeding up to two years and CFPs, including timely introduction of foods, dietary diversity, feeding frequency, hygiene, and feeding during and after illness [52]. However, it did not incorporate behavioral theory to address maternal perceptions, no counseling guide was provided, feeding assessment tools were not used, and follow-up sessions were not conducted.

### Blinding

Due to the behavioral nature of the NC intervention, blinding of participants and counselors was not feasible [53]. However, participants, counselors, and data collectors were not informed of the specific study objectives to minimize expectation bias. In addition, the data entry clerk was blinded by labeling the groups with non-identifiable codes until the analysis was completed.

### Operational definitions

Detailed operational definitions of complementary feeding indicators used in this trial have been reported previously [37].

- Timely introduction of complementary foods (TICF): Introduction of solid, semi-solid, or soft foods at 6–8 months of age [8].
- Minimum dietary diversity (MDD): Defined as consumption of foods from at least five out of eight food groups during the previous 24 hours [8].
- Minimum meal frequency (MMF): Defined as consumption of the age-appropriate minimum meal frequency per day according to WHO recommendations [8].
- Minimum acceptable diet (MAD): Defined as achievement of both minimum dietary diversity and minimum meal frequency [8].
- Egg and/or flesh food consumption (EFF): Consumption of egg and/or flesh food during the previous day [8].
- Appropriate complementary feeding practices (ACFPs): Defined as meeting all four indicators, including TICF, MDD, MMF, and EFF consumption [8].

### Measurements

HBM constructs, including perceived susceptibility, perceived severity, perceived benefits, perceived barriers, self-efficacy, and cues to action, were assessed using an instrument adapted and previously described in the baseline study [33,37]. Household wealth status and maternal knowledge and attitudes toward appropriate complementary feeding practices were also assessed using scoring procedures previously described in the same baseline study. Composite scores were computed and categorized according to predefined criteria for analysis [37].

### Data collection tools and procedures

Baseline data collection procedures and instruments used in this trial have been described previously in our baseline publications [37,38]. Baseline assessments were conducted at enrollment between June 9 and December 30, 2023, using an interviewer-administered questionnaire adapted from the 2019 Ethiopia Mini Demographic and Health Survey, the revised 2021 WHO IYCF indicators, and related literature [8,17,23,33,54]. Endline assessments were conducted between October 1–December 30, 2024, approximately 9–15 months after initiation of the intervention, depending on enrollment timing. Endline assessments included CFPs, HBM constructs, and maternal knowledge and attitudes toward complementary feeding practices. Dietary intake was assessed using a 24-hour dietary recall of foods consumed during the day preceding the interview.

### Data quality assurance

Data quality assurance procedures followed the approaches described in our previously published baseline studies [37,38]. The questionnaire was translated from English into Amharic and back-translated into English to ensure consistency. Data collectors and supervisors received training before data collection. Field supervision, completeness checks, and consistency monitoring were conducted throughout the study period. Participant confidentiality was maintained by excluding personal identifiers from the questionnaires and securely storing completed forms.

### Data management and analysis

Data were analyzed using SPSS (version 26). Analytical methods were selected based on the type of variables. Baseline sociodemographic, obstetric, and child characteristics, as well as baseline cluster level characteristics, including average cluster size and primary and secondary complementary feeding indicators (ACFPs, MDD, MMF, MAD, and EEF consumption), were summarized descriptively. HBM constructs were compared between the intervention and control groups at baseline and endline using independent samples t-tests. Generalized Estimating Equations (GEE) were employed to evaluate the intervention effect by estimating marginal odds ratio derived from the time-by-treatment interaction terms.

Unadjusted difference-in-differences (DiD) estimates (in percentage points) were computed by subtracting the absolute change in the control group from that of the intervention group. These DiD estimates are presented for descriptive purposes only and were not used for statistical inference. GEE analyses with a binomial distribution and logit link function were used to evaluate the effect of NC on primary and secondary binary outcomes, adjusting for potential confounders. The time-by-treatment (group × time) interaction term in the GEE model estimated whether changes in complementary feeding outcomes over time differed between intervention and control groups.

GEE approach was selected to account for clustering and within-subject correlation due to repeated measurements. Because only two repeated measurements (baseline and endline) were available, different working correlation structures (autoregressive, exchangeable, dependent, and unstructured) yielded identical QIC (1099.700) and QICC (1098.054) values. Therefore, the exchangeable correlation structure was selected for its simplicity and ease of interpretation.

For each outcome, the model included the time point, intervention group, and their interaction as key predictors. Covariates included in the GEE models were selected a priori based on prior literature: maternal education [20,22,55,56], child age [17,22,57,58], frequent ANC visits (≥ 4) [17,20,57–59], and household wealth index [17,37,60,61]. These variables are consistently associated with complementary feeding practices and have been treated as potential confounders in previous interventional studies [62].

To minimize model overfitting given the relatively small number of clusters (n = 30), the number of covariates was restricted in accordance with methodological recommendations [63]. Although small-sample corrections for variance estimation have been recommended for trials with limited clusters, such corrections were not applied due to software limitations. The primary outcome was analyzed using confirmatory hypothesis testing at a two-sided α = 0.05. Secondary and exploratory outcomes were analyzed without adjustment for multiple comparisons and were interpreted as supportive findings. Multicollinearity among independent variables was assessed using the variance inflation factor (VIF), which ranged from 1.005 to 1.059, indicating no significant multicollinearity. The analysis adhered to the intention-to-treat (ITT) principle, with all participants analyzed according to their originally assigned groups, regardless of adherence to the intervention. All statistical tests were two-sided with a significance level of 0.05.

### Ethical considerations

This study was conducted in accordance with the principles of the Declaration of Helsinki [64]. The trial was registered at ClinicalTrials.gov (NCT05871346), and participant flow during the trial is presented according to the CONSORT extension guidelines for cluster randomized trials [53] (Fig 1). Ethical approval was obtained from Bahir Dar University, College of Medicine and Health Sciences Institutional Review Board (IRB) (Protocol number: 703/2023). Informed consent procedures and confidentiality safeguards followed the approaches described in our previously published baseline study [37]. Written informed consent or fingerprint-confirmed consent was obtained from all mothers prior to enrollment.

## Results

### Baseline characteristics of enrolled participants

Of the 802 participants randomized, all were included in the intention-to-treat (ITT) analysis according to their originally assigned groups. Follow-up outcome data were available for 776 participants (96.8%), while 26 participants (3.2%) had no post-baseline outcome measurements. The primary analysis was conducted using Generalized Estimating Equations (GEE), which incorporate all available repeated outcome measurements without requiring complete follow-up for each participant. Participants who did not provide post-baseline data contributed their baseline measurements within the GEE framework; no randomized participants were excluded from the analysis. Attrition was low and relatively balanced between study groups (10 in the intervention group vs. 16 in the control group).

### Baseline sociodemographic, obstetric, and child characteristics

At baseline, the mean (SD) maternal age was 30.16 (5.62) years in the intervention group (IG) and 29.02 (5.74) years in the control group (CG). More than half of the mothers (53.4%) in both groups had no formal education, and the majority (97.0%) were married. Most households (82.3% IG vs.78.6% CG), reported joint decision-making on household resources.

Regarding obstetric characteristics, mothers with ≥ 2 births constituted the majority in both groups (83.0% IG vs. 75.8% CG). Most mothers had attended antenatal care, with over half (54.1% IG vs. 51.9% CG) reporting four or more visits. Nearly two-thirds of mothers (63.8% IG vs. 61.1% CG) attended postnatal care services in both groups. The sex distribution of the infants showed some imbalance between the intervention and control groups (55.4% IG vs 43.9% CG) (Table 1).

**Table 1:** Baseline sociodemographic and obstetric characteristics of mothers and their infants aged 6–8 months in West Gojjam Zone, Northwest Ethiopia.

Variable	Category	IG (n1 = 401)	CG (n2 = 401)
		Frequency (%)	Frequency (%)
Maternal age in years	Mean (SD)	30.16 (5.62)	29.02 (5.74)
Maternal education	No education	214 (53.4)	214 (53.4)
	Primary education	144 (35.9)	151 (37.7)
	Secondary/higher	43 (10.7)	36 (9.0)
Current marital status	Married (n = 778)	389 (97.0)	389 (97.0)
	Others^b^	12 (3.0)	12 (3.0)
Husband education (n = 778)	No education	120 (29.9)	117 (29.2)
	Primary education	228 (56.9)	248 (61.8)
	Secondary/higher	41 (10.2)	24 (6.0)
Decision-making on household resources	Husband	50 (12.5)	62 (15.5)
	Wife	21 (5.2)	24 (6.0)
	Jointly	330 (82.3)	315 (78.6)
Wealth index	Poorest	79 (19.7)	80 (20.0)
	Poor	75 (18.7)	87 (21.7)
	Medium	81 (20.2)	79 (19.7)
	Rich	77 (19.2)	83 (20.7)
	Richest	89 (22.2)	72 (18.0)
Maternal parity	Primipara (1)	68 (17.0)	97 (24.2)
	Multipara (2–4)	248 (61.8)	233 (58.1)
	Grand multipara (5+)	85 (21.2)	71 (17.7)
Antenatal care (ANC) visits	Yes	372 (92.8)	361 (90.0)
	No	29 (7.2)	40 (10.0)
Number of ANC visits (n = 733)	≤ 3 times	155 (38.7)	153 (38.2)
	≥ 4 times	217 (54.1)	208 (51.9)
Postnatal care visits	Yes	256 (63.8)	245 (61.1)
	No	145 (36.2)	156 (38.9)
Sex of child	Male	222 (55.4)	176 (43.9)
	Female	179 (44.6)	225 (56.1)
Age of child in months	6 months	154 (38.4)	142 (35.4)
	7 months	144 (35.9)	133 (33.2)
	8 months	103 (25.7)	126 (31.4)

ANC: Antenatal care, IG: Intervention group CG: Control group.

### Proportion of HBM construct levels between intervention and control groups

At baseline, there were no significant differences between groups across most HBM constructs, except perceived barriers. At endline, the intervention group showed significantly higher levels of perceived susceptibility, severity, benefits, cues to action, and self-efficacy, and lower perceived barriers compared with the control group (Table 2).

**Table 2:** Mean (SD) of Health Belief Model (HBM) constructs by study period and group among mothers with children aged 6–23 months in West Gojjam Zone, Northwest Ethiopia.

Health Belief Model Constructs	Study period	Intervention group, Mean (SD)	Control group, Mean (SD)	P-value
Perceived susceptibility to child undernutrition	Baseline	0.30 ± 0.46	0.27 ± 0.45	0.311
	Endline	0.44 ± 0.50	0.28 ± 0.45	< 0.001
Perceived severity of child undernutrition	Baseline	0.29 ± 0.46	0.36 ± 0.48	0.060
	Endline	0.40 ± 0.49	0.30 ± 0.46	0.007
Perceived benefits of appropriate complementary feeding	Baseline	0.35 ± 0.48	0.40 ± 0.49	0.166
	Endline	0.52 ± 0.50	0.35 ± 0.48	< 0.001
Perceived barriers to appropriate complementary feeding	Baseline	0.52 ± 0.50	0.43 ± 0.50	0.011
	Endline	0.36 ± 0.48	0.50 ± 0.50	< 0.001
Cues to action for appropriate complementary feeding	Baseline	0.32 ± 0.47	0.34 ± 0.47	0.499
	Endline	0.45 ± 0.50	0.38 ± 0.50	0.045
Self-efficacy for appropriate complementary feeding	Baseline	0.32 ± 0.47	0.31 ± 0.47	0.880
	Endline	0.45 ± 0.50	0.31 ± 0.46	< 0.001

Note: Values are presented as mean (SD) of composite HBM constructs; P-values were obtained from independent t-test.

### Baseline cluster-level complementary feeding practices

Baseline cluster-level proportions were similar between study groups, except for EFF consumption. The mean (SD) cluster size was 26.7 (4.30) mothers per cluster in the IG and 26.7 (4.13) in the CG. The primary outcome, ACFPs (10.6% vs. 8.2%), and the secondary feeding indicators, including MDD (13.8% vs. 13.1%), MMF (69.0% vs. 64.7%), and MAD (13.4% vs. 12.8%), were similar across study groups. EFF consumption was higher in the intervention clusters compared with the control clusters (27.8% vs. 19.4%). Because clusters were randomly allocated, these baseline differences are likely attributable to chance variation (Table 3).

**Table 3:** Baseline cluster-level complementary feeding practices by study arm.

Characteristic	Intervention (15 clusters)	Control (15 clusters)
Mean cluster size (SD)	26.73 (4.30)	26.73 (4.13)
Mean ACFPs prevalence (%)	10.64 (8.78)	8.23 (6.54)
Mean MDD prevalence (%)	13.82 (10.46)	13.09 (8.88)
Mean MMF prevalence (%)	69.04 (12.48)	64.66 (13.05)
Mean MAD prevalence (%)	13.40 (10.37)	12.81 (9.10)
Mean EFF consumption (%)	27.81 (3.34)	19.40 (9.23)

Note: Values are mean (SD) at the cluster level. ACFPs: Appropriate complementary feeding practices; MDD: Minimum dietary diversity; MMF: Minimum meal frequency; MAD: Minimum acceptable diet; EFF: Egg and/or flesh food.

### Changes in complementary feeding practices: A descriptive difference-in-differences analysis

At endline, ACFPs increased by 13.3 percentage points in the IG compared with 0.1 percentage points in the CG, yielding a DiD of 13.2 percentage points. MDD; ≥ 5 food groups increased by 27.7 percentage points in the IG and 12.7 percentage points in the CG, resulting in a 15.0 percentage point DiD. MMF; ≥ 3 meals per day increased by 13.6 percentage points in the IG compared with 5.8 in the CG, corresponding to a 7.8 percentage point DiD.

Among the indicators, MAD showed the largest differential improvement, increasing by 24.4 percentage points in the IG compared with 7.1 percentage points in the CG, yielding a 17.3 percentage point DiD. EFF consumption increased in both groups (22.2 vs. 19.0 percentage points), resulting in a comparatively smaller 3.2 percentage point DiD. Overall, improvements were consistently greater in the intervention group across all complementary feeding indicators (Table 4).

**Table 4:** Baseline and endline proportions and absolute difference-in-differences (percentage points) for complementary feeding indicators.

Variable	Intervention group			Control group			DiD (PP)
	Baseline (n = 401), %	Endline (n = 391), %	Difference (EL-BL), PP	Baseline (n = 401), %	Endline (n = 385), %	Difference (EL-BL), PP	
ACFPs	43 (10.7)	94 (24.0)	13.3	34 (8.5)	33 (8.6)	0.1	13.2
MDD	56 (14.0)	163 (41.7)	27.7	54 (13.5)	101 (26.2)	12.7	15.0
MMF	276 (68.8)	322 (82.4)	13.6	260 (64.8)	272 (70.6)	5.8	7.8
MAD	54 (13.5)	148 (37.9)	24.4	53 (13.2)	78 (20.3)	7.1	17.3
EFF consumption	111 (27.7)	195 (49.9)	22.2	79 (19.7)	149 (38.7)	19.0	3.2

ACFPs: Appropriate complementary feeding practices; MDD: Minimum dietary diversity; MMF: Minimum meal frequency; MAD: Minimum acceptable diet; EFF: Egg and/or flesh food; EL-BL: Endline minus baseline; PP: Percentage point; DiD: Difference-in-differences. Note: These DiD estimates are presented for descriptive interpretation and were not used for statistical inference.

### Effect of nutrition counseling on maternal knowledge and attitudes toward ACFPs

The intervention was positively associated with maternal knowledge of ACFPs. Mothers in the intervention group had higher odds of improved knowledge compared with those in the control group [AOR = 6.61, 95% CI (2.98, 14.65)]. However, the intervention was not statistically associated with maternal attitudes toward ACFPs [AOR = 1.45, 95% CI (0.99, 2.12)] (Table 5).

**Table 5:** Effect of nutrition counseling on maternal knowledge and attitudes toward appropriate complementary feeding practices.

Variable	Analysis (GEE)	β (SE)	AOR	95% CI	P-value
Knowledge on ACFPs	Multinomial cumulative logit	1.888 (0.406)	6.61	2.98, 14.65	< 0.001
Attitudes of ACFPs	Binary logistic	0.370 (0.194)	1.45	0.99, 2.12	0.056

ACFPs: Appropriate complementary feeding practices; GEE: Generalized estimating equations; β: Regression coefficient; SE: Standard error; AOR: Adjusted odds ratio; CI: Confidence interval. Note: Maternal knowledge (good, average/poor) was analyzed using multinomial cumulative logit GEE, whereas attitudes (positive/negative) were analyzed using binary logistic GEE.

### GEE analysis of the effect of nutrition counseling on complementary feeding practice indicators

While the DiD estimates presented in Table 4 describe absolute changes over time, statistical inference for the intervention effect was based on the time × group interaction term in the GEE model. After adjusting for potential confounders, the nutrition counseling intervention showed a statistically significant effect on the primary outcome (ACFPs). At endline, mothers in the intervention group were nearly 2.5 times [AOR = 2.49, 95% CI: (1.40, 4.42)] more likely to practice appropriate feeding than those in the control group. Higher household wealth was also significantly associated with ACFPs [AOR = 1.19, 95% CI: (1.04, 1.36)].

Among the secondary outcomes, children of mothers in the intervention group had higher odds of meeting MDD [AOR = 1.75, 95% CI: (1.04, 2.93)], MMF [AOR = 1.62, 95% CI: (1.01, 2.61)], and MAD [AOR = 2.13, 95% CI: (1.26, 3.59)] compared with controls. Mothers with ≥ 4 antenatal care visits had higher odds of meeting MMF [AOR = 1.28, 95% CI: (1.02, 1.63)], and higher household wealth was also significantly associated with MAD [AOR = 1.13, 95% CI: (1.02, 1.26)].

Time independently had a positive effect on EFF consumption at endline compared with baseline [AOR = 2.46, 95% CI: (1.71, 3.53)]. Independent of time, children in the intervention group were more likely to consume EFF compared with controls [AOR = 1.47, 95% CI: (1.05, 2.08)]. However, the time × group interaction for EFF consumption was not statistically significant [AOR = 1.10, 95% CI: (0.72, 1.70)], indicating that the intervention did not independently improve EFF consumption beyond temporal increases observed in both study groups. Although baseline EFF consumption was higher in the intervention group (Table 3), this imbalance was accounted for in the longitudinal GEE model and did not materially influence the estimated intervention effect. Other covariates, including maternal education and child age, were not significantly associated with any of the outcomes (p > 0.05) (Table 6).

**Table 6:** GEE analysis of the effect of nutrition counseling on complementary feeding practice indicators.

Outcome	Variable	Beta coefficient	Standard error	95% CI for β	P-value	AOR	95% CI for AOR
ACFPs	Time	0.083	0.2540	(−0.415, 0.581)	0.744	1.086	(0.660, 1.787)
	Group	0.190	0.2494	(−0.299, 0.679)	0.447	1.209	(0.742, 1.971)
	Time× group	0.913	0.2927	(0.339, 1.487)	0.002	2.492	(1.404, 4.423)
	Wealth index	0.172	0.0689	(0.037, 0.307)	0.013	1.187	(1.037, 1.359)
MDD	Time	0.896	0.2184	(0.468, 1.324)	< 0.001	2.450	(1.597, 3.759)
	Group	0.091	0.2149	(−0.330, 0.512)	0.671	1.096	(0.719, 1.669)
	Time× group	0.557	0.2642	(0.039, 1.075)	0.035	1.745	(1.040, 2.929)
MMF	Time	0.229	0.1907	(−0.145, 0.603)	0.230	1.257	(0.865, 1.827)
	Group	0.140	0.1578	(−0.169, 0.450)	0.373	1.151	(0.845, 1.568)
	Time× group	0.485	0.2414	(0.012, 0.958)	0.045	1.624	(1.012, 2.607)
	ANC (≥ 4)	0.250	0.1201	(0.014, 0.485)	0.037	1.284	(1.015, 1.625)
MAD	Time	0.655	0.2233	(0.217, 1.093)	0.003	1.925	(1.243, 2.982)
	Group	0.066	0.2175	(−0.361, 0.492)	0.763	1.068	(0.697, 1.636)
	Time× group	0.754	0.2671	(0.230, 1.278)	0.005	2.126	(1.259, 3.588)
	Wealth index	0.124	0.0524	(0.022, 0.227)	0.018	1.132	(1.022, 1.255)
EFF consumption	Time	0.899	0.1848	(0.537, 1.261)	< 0.001	2.458	(1.711, 3.530)
	Group	0.388	0.1751	(0.045, 0.731)	0.027	1.474	(1.046, 2.078)
	Time× group	0.098	0.2210	(−0.335, 0.531)	0.657	1.103	(0.715, 1.701)

The model was adjusted for maternal education, wealth index, child age, and antenatal care visits (≥ 4).

ACFPs: Appropriate complementary feeding practices, MDD: Minimum dietary diversity, MMF: Minimum meal frequency, MAD: Minimum acceptable diet, EFF: Eggs and/or flesh foods, β: Regression coefficient**;** AOR: Adjusted odds ratio, CI: confidence interval; ANC (≥ 4): Antenatal care visits ≥ 4.

## Discussion

This trial found that an HBM-based NC intervention was associated with significant improvements in appropriate complementary feeding practices among mothers with children aged 6–23 months. These findings add to the existing evidence by demonstrating the effect of HBM-based NC in a cluster-randomized controlled trial conducted in a rural Ethiopian setting. The study integrated behavioral theory with longitudinal assessment of both behavioral determinants and feeding outcomes. ACFPs were evaluated as a composite indicator of key WHO feeding recommendations, providing a more comprehensive assessment of feeding adequacy.

In this study, ACFPs were defined by simultaneous fulfillment of TICF, MDD, MMF, and EFF consumption. While this definition is rigorous and aligns with current WHO recommendations for optimal complementary feeding, it is more stringent than definitions used in some previous studies that rely on fewer indicators. As a result, the absolute percentage of ACFPs observed in this study may appear modest. However, this definition ensures that children meet comprehensive dietary adequacy rather than partial compliance with selected indicators.

The proportion of mothers practicing appropriate complementary feeding was higher in the intervention group than in the control group. After adjusting for potential confounders, the GEE analysis showed that the intervention was associated with higher odds of ACFPs. These findings are consistent with previous studies. A nutrition education intervention conducted in daycare centers in Hawassa Town, Southern Ethiopia, similarly reported improved CFPs among mothers in the intervention group compared with the control group [36]. Likewise, a community-based NC intervention in rural Uttar Pradesh, India, substantially improved CFPs, as demonstrated by increases in complementary feeding index scores among intervention mothers [65].

Improvements in feeding practices were accompanied by increases in HBM constructs. This pattern aligns with findings from Ghirokarzin City, Fars Province, Iran, where HBM-based NC improved mothers’ HBM constructs and complementary feeding behaviors [66]. These results support the notion that theory-based counseling may promote behavioral change by improving maternal knowledge, perceived benefits, and self-efficacy while reducing perceived barriers. As mothers became more aware that inadequate dietary diversity and meal frequency during this critical developmental period could adversely affect their children’s nutritional outcomes, they became more motivated to practice appropriate complementary feeding.

The improvements observed in our study may also be attributed to the delivery approach. Counseling was delivered monthly at participants’ homes using a client-centered approach in the local language. The intervention began when infants were 6–8 months old and used counseling guides featuring simple, illustrated messages. Before each session, mothers’ feeding practices were assessed using the Food Intake–6–24 Months Job Aid.

Counseling then focused on two or three priority topics identified through these assessments. The use of age-appropriate counseling cards with clear illustrations likely facilitated information retention and supported improvements in feeding behaviors. These materials were designed to promote sustained adoption of recommended feeding practices.

In addition to the NC intervention, household socioeconomic conditions also played a role. Higher household wealth was positively associated with ACFPs, consistent with cross-sectional studies in Ethiopia [17,37,60]. This association may reflect greater purchasing power among wealthier households, facilitating dietary diversification and adoption of recommended feeding practices.

Beyond the primary outcome, improvements were also observed in individual complementary feeding indicators, including MDD, MMF, and MAD. The proportion of children achieving MDD was higher in the intervention group than in the control group. This difference remained significant after controlling for potential confounders. Similar improvements have been reported in Dore Bafano district, Southern Ethiopia, where HBM-based education enhanced child dietary diversity [30]. A positive effect has also been observed in other settings, including Ethiopia [44,67], Tanzania, Bangladesh, and China [68–70], underscoring that NC is effective when adapted to local sociocultural contexts.

A cluster-randomized trial in West Gojjam Zone reported that behavior change communication intervention was associated with increased likelihood of achieving MDD (RR = 3.0), MMF (RR = 2.4), and MAD (RR = 2.7) [44]. The associations observed in our study (AORs 1.62–2.49) are within the range of these reported effect sizes, suggesting that behavior change interventions, including theory-based counseling, are associated with improvements in CFPs.

In this study, children of mothers in the intervention group had higher odds of meeting MMF compared with controls. Similar improvements have been reported in Ethiopia [44], as well as in rural Tanzania, Bangladesh, and China [68–70]. This improvement may reflect repeated exposure to nutrition counseling, which likely motivated behavior change. Additionally, mothers who attended ≥ 4 antenatal care visits had higher odds of meeting MMF for their children compared with those with fewer visits. Likewise, in South, East, and Central regions of India, attending ≥ 4 antenatal visits was significantly associated with improved MMF [59]. These findings underscore frequent antenatal visits as an important opportunity for promoting recommended child feeding practices.

The proportion of children meeting the MAD was also higher in the intervention group than in the control group. The GEE analysis showed that children in the intervention group were more likely to achieve MAD than those in the control group. Similar improvements have been observed in theory-based interventions in Southern Ethiopia [67] and a behavior change communication intervention in rural West Gojjam Zone, Northwest Ethiopia, which reported significant gains in children’s MAD [44]. In addition, higher household wealth was positively associated with achieving MAD, consistent with evidence from cross-sectional studies in Ethiopia and other parts of sub-Saharan Africa [61,71], likely reflecting the greater ability of wealthier households to provide diverse and frequent meals.

Although baseline EFF consumption differed between study groups, this likely reflects chance variation inherent in cluster randomization. GEE analysis accounted for these baseline differences through inclusion of time-by-group interaction terms. The findings indicated that the intervention did not independently improve EFF consumption and that the baseline imbalance did not materially affect the intervention effect. The limited effect may reflect that provision of EFF requires adequate household resources and stable access, which counseling alone may not sufficiently address [72]. Cultural norms and household decision-making dynamics may also constrain mothers’ ability to adopt these practices, despite increased awareness [73]. Additionally, substantial improvements in EEF consumption in the control group over time (19.7% at baseline vs. 38.7% at endline) may have reduced the between-group difference.

While the intervention significantly improved maternal knowledge of ACFPs, it did not result in a statistically significant change in attitudes. Knowledge is generally more responsive to counseling, whereas attitudes are more strongly influenced by household decision-making dynamics and cultural beliefs. Similar findings have been reported in Indonesia, where improvements in maternal knowledge were not accompanied by corresponding changes in attitudes or feeding practices [74]. The borderline significance for attitudes (P = 0.056) suggests that more intensive community-based behavior change strategies may be needed to achieve sustained behavioral change. These may include male partner engagement, social norm interventions, and improved household access to affordable animal-source foods.

**Strengths and limitations:** This cluster-randomized controlled trial employed a theory-driven counseling approach supported by illustrated guides and assessed primary and secondary complementary feeding outcomes. The use of descriptive DiD estimates and GEE analyses for confirmatory inference strengthened internal validity. However, dietary intake data were self-reported and may be subject to recall and social desirability bias. Blinding of participants and data collectors to specific study objectives was not feasible due to the nature of the intervention, which may have introduced reporting bias. The trial was conducted only in rural settings, limiting generalizability to urban populations. In addition, the NC intervention alone may have limited capacity to modify household economic constraints, particularly regarding access to animal-source foods. To maintain model stability, covariate adjustment was limited; nevertheless, the relatively small number of clusters may have reduced the precision of variance estimates, and small-sample corrections were not applied because of software limitations. Secondary and exploratory outcomes were analyzed without adjustment for multiple comparisons and should be interpreted as supportive findings. Baseline EFF consumption differed between groups; however, this was accounted for in the longitudinal analysis and did not materially alter the estimated intervention effect.

## Conclusion

This trial provides evidence that an HBM-based NC significantly improved complementary feeding practices among mothers with children aged 6–23 months. The intervention improved ACFPs and key secondary outcomes, including MDD, MMF, and MAD, and positively influenced behavioral determinants such as perceived benefits, reduced perceived barriers, and enhanced self-efficacy. These findings suggest that integrating illustrated, home-based NC into routine maternal and child health services, including antenatal and postnatal care platforms, may strengthen child nutrition programs and support efforts to reduce undernutrition in similar rural settings. Future research should evaluate scalability and cost-effectiveness in comparable low-resource contexts.

## Supporting information

S1 Appendix CONSORT checklist. (DOCX)
