# Supplementary material for: Effect of nutrition counseling on complementary feeding practices among mothers with children aged 6–23 months in West Gojjam Zone, Northwest Ethiopia: A cluster-randomized controlled trial
Source: PLoS One. 2026 Oct 29;21(10):e0361976. doi: 10.1371/journal.pone.0361976 (PMC13718931; doi:10.1371/journal.pone.0361976)
Supplement: Supplementary file 1 [file pone.0361976.s001.docx]

**Table 1: CONSORT 2010 checklist of information to include when reporting a cluster randomised trial**

| **Section/Topic** | **Item No** | **Standard Checklist item** | | **Page (s) & Line (s)** | **Extension for cluster designs** | **Page (s) & Line (s)** |
| --- | --- | --- | --- | --- | --- | --- |
| Title & abstract |  |  | |  |  |  |
|  | 1a | Identification as a randomised trial in the title | | 1, lines 1-3 | Identification as a cluster randomised trial in the title | 1, lines 1-3 |
|  | 1b | Structured summary of trial design, methods, results, and conclusions (for specific guidance see CONSORT for abstracts)^[1],^^[2]^ | | 2-3, lines 21-52 | See table 2 |  |
|  |  |  | |  |  |  |
|  | | | Introduction | | |  |
| Background and objectives | 2a | Scientific background and explanation of rationale | | 4-6, lines 66-121 | Rationale for using a cluster design | 4-6, lines 66-121 |
|  | 2b | Specific objectives or hypotheses | | 6, lines 119-121 | Whether objectives pertain to the cluster level, the individual participant level or both | 6, lines 119-121 |
|  | | | **Methods** | | |  |
| Trial design | 3a | Description of trial design (such as parallel, factorial) including allocation ratio | | 6, lines 127-136 & 10-11, lines 210-241 | Definition of cluster and description of how the design features apply to the clusters | 6, lines 127-136 & 10-11, lines 210-241 |
|  | 3b | Important changes to methods after trial commencement (such as eligibility criteria), with reasons | | No changes in eligibility criteria |  |  |
| Participants | 4a | Eligibility criteria for participants | | 8, lines 161-165 | Eligibility criteria for clusters | 10-11, lines 210-241 |
|  | 4b | Settings and locations where the data were collected | | 6, lines 123-126 |  |  |
| Interventions | 5 | The interventions for each group with sufficient details to allow replication, including how and when they were administered | | 11-14, lines 245-305 | Whether interventions pertain to the cluster level, the individual participant level or both | 11-14, lines 245-305 |
| Outcomes | 6a | Completely defined pre-specified primary and secondary outcome measures, including how and when they were assessed | | 9, lines 181-190 | Whether outcome measures pertain to the cluster level, the individual participant level or both | 9, lines 181-190 |
|  | 6b | Any changes to trial outcomes after the trial commenced, with reasons | | No changes made, but primary and secondary outcomes are explicitly described |  |  |
| Sample size | 7a | How sample size was determined | | 9-10, lines 191-209 | Method of calculation, number of clusters(s) (and whether equal or unequal cluster sizes are assumed), cluster size, a coefficient of intra-cluster correlation (ICC or *k*), and an indication of its uncertainty | 9-10, lines 191-209 |
|  | 7b | When applicable, explanation of any interim analyses and stopping guidelines | | Not applicable. |  |  |
|  | | | **Randomisation:** | | |  |
| Sequence generation | 8a | Method used to generate the random allocation sequence | | 10-11, lines 210-244 |  |  |
|  | 8b | Type of randomisation; details of any restriction (such as blocking and block size) | | 10-11, lines 210-244 | Details of stratification or matching if used | 10-11, lines 210-244 |
| Allocation concealment mechanism | 9 | Mechanism used to implement the random allocation sequence (such as sequentially numbered containers), describing any steps taken to conceal the sequence until interventions were assigned | | 11, lines 235-241 | Specification that allocation was based on clusters rather than individuals and whether allocation concealment (if any) was at the cluster level, the individual participant level or both | 11, lines 235-241 |
| Implementation | 10 | Who generated the random allocation sequence, who enrolled participants, and who assigned participants to interventions | | 11, lines 235-241 | Replace by 10a, 10b and 10c |  |
|  | 10a |  | |  | Who generated the random allocation sequence, who enrolled clusters, and who assigned clusters to interventions | 11, lines 235-241 |
|  | 10b |  | |  | Mechanism by which individual participants were included in clusters for the purposes of the trial (such as complete enumeration, random sampling) | 10, lines 213-216 |
|  | 10c |  | |  | From whom consent was sought (representatives of the cluster, or individual cluster members, or both), and whether consent was sought before or after randomisation | 18, lines 196-399 |
|  |  |  | |  |  |  |
| Blinding | 11a | If done, who was blinded after assignment to interventions (for example, participants, care providers, those assessing outcomes) and how | | 14, lines 306-310 |  |  |
|  | 11b | If relevant, description of the similarity of interventions | |  |  |  |
| Statistical methods | 12a | Statistical methods used to compare groups for primary and secondary outcomes | | 16-18, lines 351-390 | How clustering was taken in to account | 16-18, lines 351-390 |
|  | 12b | Methods for additional analyses, such as subgroup analyses and adjusted analyses | | 16-18, lines 351-390 |  |  |
|  | | | **Results** | | |  |
| Participant flow (a diagram is strongly recommended) | 13a | For each group, the numbers of participants who were randomly assigned, received intended treatment, and were analysed for the primary outcome | | Assigned: 8, lines 171-180 (see Fig1) | For each group, the numbers of clusters that were randomly assigned, received intended treatment, and were analysed for the primary outcome | Assigned: 8, lines 171-180 (see Fig1) |
|  | 13b | For each group, losses, and exclusions after randomisation, together with reasons | | Assigned: 8, lines 171-180 (see Fig1) | For each group, losses and exclusions for both clusters and individual cluster members | Assigned: 8, lines 171-180 (see Fig1) |
| Recruitment | 14a | Dates defining the periods of recruitment and follow-up | | 6, line 132-136 |  |  |
|  | 14b | Why the trial ended or was stopped | | Study completed. |  |  |
| Baseline data | 15 | A table showing baseline demographic and clinical characteristics for each group | | 18-20, lines 401-429 | Baseline characteristics for the individual and cluster levels as applicable for each group | 18-20, lines 401-429 |
| Numbers analysed | 16 | For each group, number of participants (denominator) included in each analysis and whether the analysis was by original assigned groups | | 18-20, lines 401-429 | For each group, number of clusters included in each analysis | 18-20, lines 401-429 |
| Outcomes and estimation | 17a | For each primary and secondary outcome, results for each group, and the estimated effect size and its precision (such as 95% confidence interval) | | 25-28, lines 478-507 | Results at the individual or cluster level as applicable and a coefficient of intra-cluster correlation (ICC or k) for each primary outcome | 25-28, lines 478-507 |
|  | 17b | For binary outcomes, presentation of both absolute and relative effect sizes is recommended | | 25-26, lines 482-499 & 25-28, lines 478-507 |  |  |
| Ancillary analyses | 18 | Results of any other analyses performed, including subgroup analyses and adjusted analyses, distinguishing pre-specified from exploratory | | Adjusted analyses (25-28, lines 478-507) |  |  |
| Harms | 19 | All-important harms or unintended effects in each group (for specific guidance see CONSORT for harms^[3]^) | | No harms. |  |  |
|  | | | **Discussion** | | |  |
| Limitations | 20 | Trial limitations, addressing sources of potential bias, imprecision, and, if relevant, multiplicity of analyses | | 33, lines 606-618 |  |  |
| Generalisability | 21 | Generalisability (external validity, applicability) of the trial findings | | 33, lines 609-610 | Generalisability to clusters and/or individual participants (as relevant) | 33, lines 609-610 |
| Interpretation | 22 | Interpretation consistent with results, balancing benefits and harms, and considering other relevant evidence | | 29-33, lines 509-602 |  |  |
| **Other information** | | | |  |  |  |
| Registration | 23 | Registration number and name of trial registry | | 3, line 51 |  |  |
| Protocol | 24 | Where the full trial protocol can be accessed, if available | | Uploaded as supporting information (page 6, line 128, S1 Appendix.) |  |  |
| Funding | 25 | Sources of funding and other support (such as supply of drugs), role of funders | | 34, lines 635-637 |  |  |

** Note: page numbers optional depending on journal requirements*

**Table 2:	Extension of CONSORT for abstracts**1**^,^**2 **to reports of cluster randomised trials**

| **Item** | **Standard Checklist item** | **Page (s) & line (s)** | **Extension for cluster trials** | **Page (s) & line (s)** |
| --- | --- | --- | --- | --- |
| Title | Identification of study as randomised | 1, lines 1-3 | Identification of study as cluster randomised | 1, lines 1-3 |
| Trial design | Description of the trial design (e.g. parallel, cluster, non-inferiority) | 2, lines 28-29 |  |  |
| **Methods** |  |  |  |  |
| Participants | Eligibility criteria for participants and the settings where the data were collected | 2, lines 24-26 & 32-33 | Eligibility criteria for clusters | 2, lines 29-32 |
| Interventions | Interventions intended for each group | 2, lines 29-32 |  |  |
| Objective | Specific objective or hypothesis | 2, lines 24-26 | Whether objective or hypothesis pertains to the cluster level, the individual participant level or both | 2, lines 24-26 |
| Outcome | Clearly defined primary outcome for this report | 2, lines 33-35 | Whether the primary outcome pertains to the cluster level, the individual participant level or both | 2, lines 33-35 |
| Randomization | How participants were allocated to interventions | 2, lines 29-33 | How clusters were allocated to interventions | 2, lines 29-33 |
| Blinding (masking) | Whether or not participants, care givers, and those assessing the outcomes were blinded to group assignment |  |  |  |
| **Results** |  |  |  |  |
| Numbers randomized | Number of participants randomized to each group | 2, lines 30-33 | Number of clusters randomized to each group | 2, line 29 |
| Recruitment | Trial status^[4]^ | **Completed** |  |  |
| Numbers analysed | Number of participants analysed in each group | 2, lines 29-32 | Number of clusters analysed in each group | 2, line 29 |
| Outcome | For the primary outcome, a result for each group and the estimated effect size and its precision | 2-3, lines 38-44 | Results at the cluster or individual participant level as applicable for each primary outcome | 2-3, lines 38-44 |
| Harms | Important adverse events or side effects | No harms. |  |  |
| Conclusions | General interpretation of the results | 3, lines 45-49 |  |  |
| Trial registration | Registration number and name of trial register | 3, line 51 |  |  |
| Funding | Source of funding | 3, line 52 |  |  |

**REFERENCES**

1. Hopewell S, Clarke M, Moher D, Wager E, Middleton P, Altman DG, et al. CONSORT for reporting randomized trials in journal and conference abstracts. *Lancet* 2008, 371:281-283
2. Hopewell S, Clarke M, Moher D, Wager E, Middleton P, Altman DG at al (2008) CONSORT for reporting randomized controlled trials in journal and conference abstracts: explanation and elaboration. *PLoS Med* 5(1): e20
3. Ioannidis JP, Evans SJ, Gotzsche PC, O'Neill RT, Altman DG, Schulz K, Moher D. Better reporting of harms in randomized trials: an extension of the CONSORT statement. *Ann Intern Med* 2004; 141(10):781-788.
4. Relevant to Conference Abstracts
